# Continuous Monitoring of Cerebral Autoregulation in Adults Supported by Extracorporeal Membrane Oxygenation

**DOI:** 10.21203/rs.3.rs-3300834/v1

**Published:** 2023-09-21

**Authors:** Lucy Q Zhang, Henry Chang, Andrew Kalra, Mariyam Humayun, Kathryn R Rosenblatt, Vishank A Shah, Romergryko G Geocadin, Charles H Brown, Bo Soo Kim, Glenn J.R. Whitman, Lucia Rivera-Lara, Sung-min Cho

**Affiliations:** Johns Hopkins School of Medicine: The Johns Hopkins University School of Medicine; Johns Hopkins School of Medicine: The Johns Hopkins University School of Medicine; Johns Hopkins School of Medicine: The Johns Hopkins University School of Medicine; Johns Hopkins School of Medicine: The Johns Hopkins University School of Medicine; Johns Hopkins School of Medicine: The Johns Hopkins University School of Medicine; Johns Hopkins School of Medicine: The Johns Hopkins University School of Medicine; Johns Hopkins School of Medicine: The Johns Hopkins University School of Medicine; Johns Hopkins School of Medicine: The Johns Hopkins University School of Medicine; Johns Hopkins School of Medicine: The Johns Hopkins University School of Medicine; Johns Hopkins School of Medicine: The Johns Hopkins University School of Medicine; Stanford University Department of Neurology and Neurological Sciences; Johns Hopkins Department of Anesthesiology and Critical Care Medicine: Johns Hopkins Medicine Department of Anesthesiology and Critical Care Medicine

**Keywords:** Extracorporeal membrane oxygenation, ECMO, cerebral autoregulation, optimal perfusion pressure, near-infrared spectroscopy, neurological outcomes

## Abstract

**Background:**

Impaired cerebral autoregulation (CA) is one of several proposed mechanisms of acute brain injury in patients supported by extracorporeal membrane oxygenation (ECMO). The primary aim of this study was to determine the feasibility of continuous CA monitoring in adult ECMO patients. Our secondary aims were to describe changes in cerebral oximetry index (COx) and other metrics of CA over time and in relation to functional neurologic outcomes.

**Methods:**

This is a single-center prospective observational study. We measured Cox, a surrogate measurement of cerebral blood flow, measured by near-infrared spectroscopy, which is an index of CA derived from the moving correlation between mean arterial pressure and slow waves of regional cerebral oxygen saturation. A COx value that approaches 1 indicates impaired CA. Using COx, we determined the optimal MAP (MAP_OPT_), lower and upper limits of autoregulation for individual patients. These measurements were examined in relation to modified Rankin Scale (mRS) scores.

**Results:**

Fifteen patients (median age=57 years [IQR=47–69]) with 150 autoregulation measurements were included for analysis. Eleven were on veno-arterial ECMO and 4 on veno-venous. Mean COx was higher on post-cannulation day 1 than on day 2 (0.2 vs 0.09, *p*<0.01), indicating improved CA over time. COx was higher in VA-ECMO patients than in VV-ECMO (0.12 vs 0.06, *p*=0.04). Median MAP_OPT_ for entire cohort was highly variable, ranging 55–110 mmHg. Patients with mRS 0–3 (good outcome) at 3 and 6 months spent less time outside of MAP_OPT_ compared to patients with mRS 4–6 (poor outcome) (74% vs 82%, *p*=0.01). The percentage of time when observed MAP was outside the limits of autoregulation was higher on post-cannulation day 1 than on day 2 (18.2% vs 3.3%, p<0.01).

**Conclusions:**

In ECMO patients, it is feasible to monitor CA continuously at the bedside. CA improved over time, most significantly between post-cannulation days 1 and 2. CA was more impaired in VA-ECMO than VV-ECMO. Spending less time outside of MAP_OPT_ may be associated with achieving a good neurologic outcome.

## Introduction

Extracorporeal membrane oxygenation (ECMO) provides mechanical circulatory support for critically ill patients with cardiac or respiratory failure. Neurologic complications have been reported in 10–50% of patients on ECMO support, but they remain clinically underrecognized.^1–3^ Multimodality neurologic monitoring has been proposed to aid in the detection of neurologic complications, particularly during periods of deep sedation and paralysis.^4–6^ Several mechanisms of acute brain injury in ECMO patients have been described, including impaired cerebral autoregulation.^7–9^

Cerebral autoregulation (CA) is a physiologic compensatory response aimed to maintain stable cerebral blood flow (CBF) over a range of cerebral perfusion pressure or arterial blood pressure (ABP). The integrity of CA can be inferred from several indices of CA. Cerebral oxygenation index (COx) is one index of CA that is derived from the moving correlation between slow waves of regional cerebral oxygen saturation (rSO_2_) and mean arterial pressure (MAP), a validated surrogate measurement of CBF measured by near-infrared spectroscopy (NIRS).^10–12^ NIRS has been widely used to monitor cerebral autoregulation in patients during cardiopulmonary bypass.^13–15^

In this pilot study, we aimed to determine the feasibility of continuous CA monitoring in adult patients supported by ECMO in the intensive care unit. Our secondary aims were to describe changes in COx and other metrics of CA over time and in relation to functional neurologic outcomes.

## Methods

### Study design

This is a prospective observational cohort study that was conducted at a tertiary academic hospital between January 2021 and April 2022. The study protocol was approved by the local institutional review board.

### Patients and data collection

We included adults (≥18 years old) who underwent veno-arterial (VA) or veno-venous (VV) ECMO cannulation. Patients were treated according to standard institutional practices regarding cannulation site selection, management of membrane oxygenator and circuitry, sedation, and anticoagulation strategies. We excluded patients with active SARS-CoV-2 infection who were in contact isolation and those who did not meet patient monitoring requirements (see under Cerebral autoregulation monitoring and assessment).

We collected data on demographics, medical comorbidities, and ECMO-related variables. We recorded clinical outcomes including length of stay in the intensive care unit (ICU), death at time of hospital discharge, neurological complications, and functional neurological outcomes at 3 and 6 months. Neurological complications were defined a priori as follows: 1) acute ischemic infarction 2) intracranial hemorrhage, 3) hypoxic-ischemic brain injury, 4) clinical or electrographic seizures, and 5) brain death. A good neurologic outcome is defined as a score of 0–3 on the modified Rankin Scale (mRS). Functional neurologic outcomes at 3 and 6 were obtained from outpatient clinic notes or based on documentation from rehabilitation services.

### Cerebral autoregulation monitoring and assessment

CA monitoring started within 72 hours of ECMO initiation, and the total duration of CA monitoring was at least 12 hours. CA was assessed using COx, a continuous, moving Pearson’s correlation coefficient between spontaneous variations in MAP and slow waves of rSO_2_ derived from NIRS. COx values were calculated by ICM+ software (University of Cambridge, Cambridge Enterprise Ltd, Cambridge, UK) using 10-second mean values of MAP and rSO_2_ from a 300-second window, incorporating 30 data points. An indwelling catheter in the radial or femoral artery was used to continuously record MAP. For continuous rSO_2_ monitoring, we used a NIRS device (INVOS^™^ 5100 C, Medtronic^®^, US) and placed self-adhesive sensors on the right and left side of the forehead. MAP and rSO_2_ signals were filtered by ICM+ software to eliminate high-frequency signal noise produced by respiration and pulse waveforms. This method of filtering also allows for the detection of slow-wave oscillations that occur below 0.05 Hz.^13,14,19^ COx values were averaged for the entire duration of recording as well as 6 and 24-hour intervals. When CA is intact, there is no correlation between MAP and rSO_2_, and COx approaches 0 or negative values. When COx value approaches 1, the relationship between MAP and rSO_2_ becomes passive, indicating impaired CA. Although an absolute COx cutoff for CA impairment has not been firmly established, COx value of 0.3–0.35 has been conventionally used to define the autoregulatory threshold.^16–18^ This study used COx <0.3.

### Optimal MAP, delta MAP, lower and upper limits of cerebral autoregulation

We determined optimal MAP (MAP_OPT_) values for each individual patient at 6-hour intervals by placing MAP values in 5 mmHg bins and identifying the MAP associated with the lowest COx.^19–21^ We then determined the lower limit of autoregulation (LLA), which was defined as the lowest MAP value at which COx increased from <0.3 to ≥0.3.^15,22^ Accordingly, the upper limit of autoregulation (ULA) was defined as the highest MAP value at which COx crossed the 0.3 threshold. We calculated the percentage of time the observed MAP (MAP_OBS_) was below and above MAP_OPT_, as well as the percentage of time MAP_OBS_ was below LLA and above ULA. To quantify the relationship between neurological outcome and time spent outside of LLA and ULA, we calculated the area under the curve (AUC) using the magnitude of MAP deviation (in mmHg) and duration of time (in hours).^16^

### Neurologic monitoring protocol

In addition to CA monitoring, all patients were evaluated by the neurocritical care consultation team and had serial neurologic examinations per institutional protocol. The protocol included electroencephalography (EEG), transcranial doppler, computerized tomography of the brain at the discretion of the clinician, and/or magnetic resonance imaging after decannulation. CA monitoring data were not available to the bedside clinician to guide or bias medical management.

### Statistical analysis

Quantitative patient variables were reported as medians (interquartile range: IQR) and qualitative variables as absolute frequencies in percentages. The relationship between clinical outcomes and COx along with other metrics of CA was explored graphically using scatterplots. Intergroup comparisons were made using Fisher’s exact test for categorical variables and Mann-Whitney U test for continuous variables.

We used mixed-effects models with random intercepts to compare COx on different days of monitoring and ECMO mode. The association between percentage of time spent outside the limits of autoregulation and day of monitoring was also assessed using this method. Models with an independent within-subject residual structure was used as it was best supported by the data among other correlation models considered. The method of Generalized Estimating Equations was used to examine functional outcomes at 3 and 6 months in relation to the percentage of time MAP_OBS_ was below or above the MAP_OPT_. All analyses were two-tailed, and significance level was determined by *p* value <0.05. Statistical analysis was performed using STATA 17 (StataCorp, College Station, TX, USA).

## Results

### Baseline characteristics

Fifteen (median age=57 years [IQR=47–69]; 33.3% female) of 21 enrolled patients were included in our analysis. Six patients were excluded for failing to meet patient monitoring requirements (i.e. duration of monitoring <12 hours, loss of blood pressure or rSO_2_ signal, corrupted files). A total of 150 measurements of autoregulation were included in the analysis (extracted every 6 hours over the duration of monitoring). Demographics, comorbidities, and ECMO characteristics are shown in Table 1. Seventy-three percent of patients (n=11) were supported by VA-ECMO and 27% (n=4) by VV-ECMO. Among VA-ECMO patients, six (54.5%) were peripherally cannulated, and five patients (45.5%) were centrally cannulated. Additional mechanical circulatory support devices were used in 81.8% of VA-ECMO patients (n=9). Three of the 4 VV-ECMO (75%) patients had a dual-lumen cannula. The most common ECMO indication was cardiogenic shock (n=7, 46.7%), followed by refractory respiratory failure (n=4, 26.6%), and then post-cardiotomy (n=3, 20%). One VA-ECMO patient (6.7%) was cannulated for both cardiogenic shock and respiratory failure. The median duration of ECMO support was 5 days (3–10.5) in the VA-ECMO group and 11 days (9.5–13) in VV-ECMO. Overall, the median ICU length of stay was 13 days (9.5–20). Fifty-three percent of the cohort (8/15) died before hospital discharge.

When comparing VA- and VV-ECMO patients, we observed a higher frequency of sepsis in the VV-ECMO group than VA-ECMO (100% vs 27.3%, *p*=0.03). The frequency of SARS-CoV-2 infection was also higher in the VV-ECMO group than VA-ECMO (75% vs 9.1%, *p*=0.03). VV-ECMO patients had a higher median PCO_2_ (68 mmHg [58–76] vs 38 mmHg [35–48], *p*=0.04) and lower PaO_2_ (65 mmHg [54–72] vs 132 [95–299], *p*=0.02) than VA-ECMO patients on their arterial blood gas immediately prior to cannulation. There was no difference between VA- and VV-ECMO patients in any of the prespecified clinical outcomes that this study examined.

### Cerebral autoregulation

**Figure 1.**
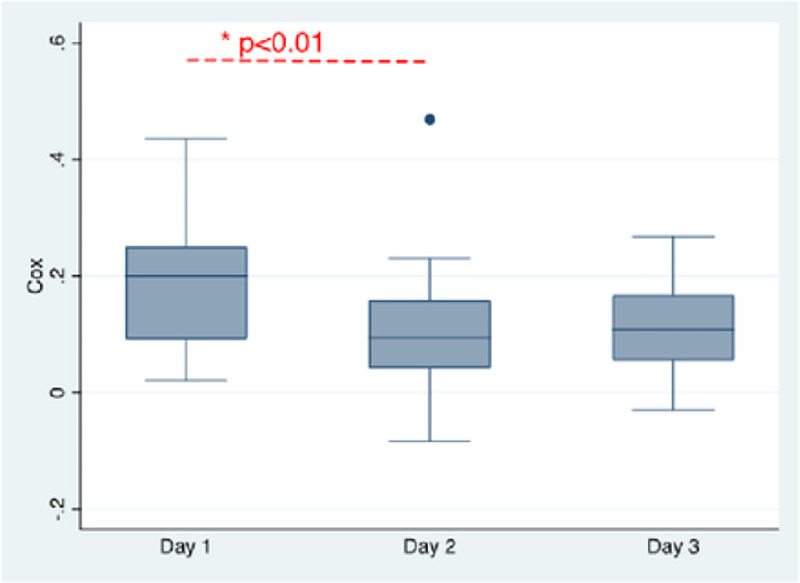
Boxplot showing the evolution of cerebral oximetry index (COx) of the entire cohort from post-cannulation day 1 to day 3.

**Figure 2.**
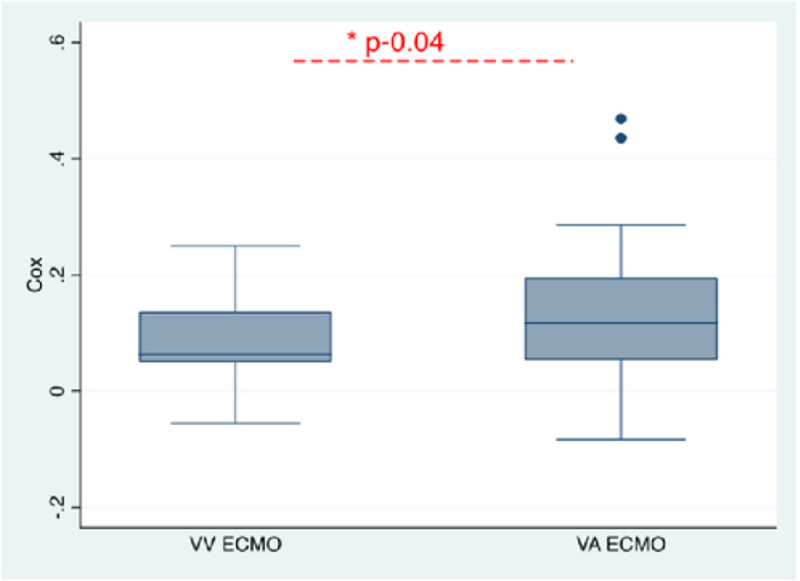
Boxplot comparing COx of veno-venous and veno-arterial ECMO patients

CA monitoring started at a median 22 hours (16–27) after ECMO cannulation. No interference with ECMO circulation or clinical care was reported. All patients tolerated continuous MAP and rSO_2_ monitoring without complications. The evolution of COx from post-cannulation day 1 to day 3 for the entire cohort is shown in Figure 1. Mean COx decreased over time and was significantly lower on post-cannulation day 2 than on day 1 (0.09 vs 0.2, *p*<0.01). Mean COx for the entire duration of neuromonitoring was significantly higher and more dysregulated in VA-ECMO patients than VV-ECMO (0.12 vs 0.06, *p*=0.04) (Figure 2).

**Figure 3.**
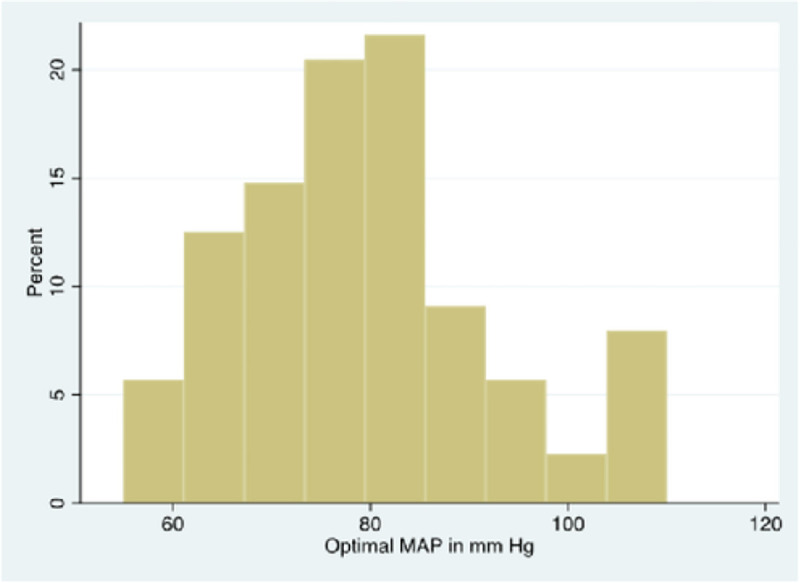
Histogram showing the percentage of time spent at different optimal MAP values for the entire cohort.

**Figure 4.**
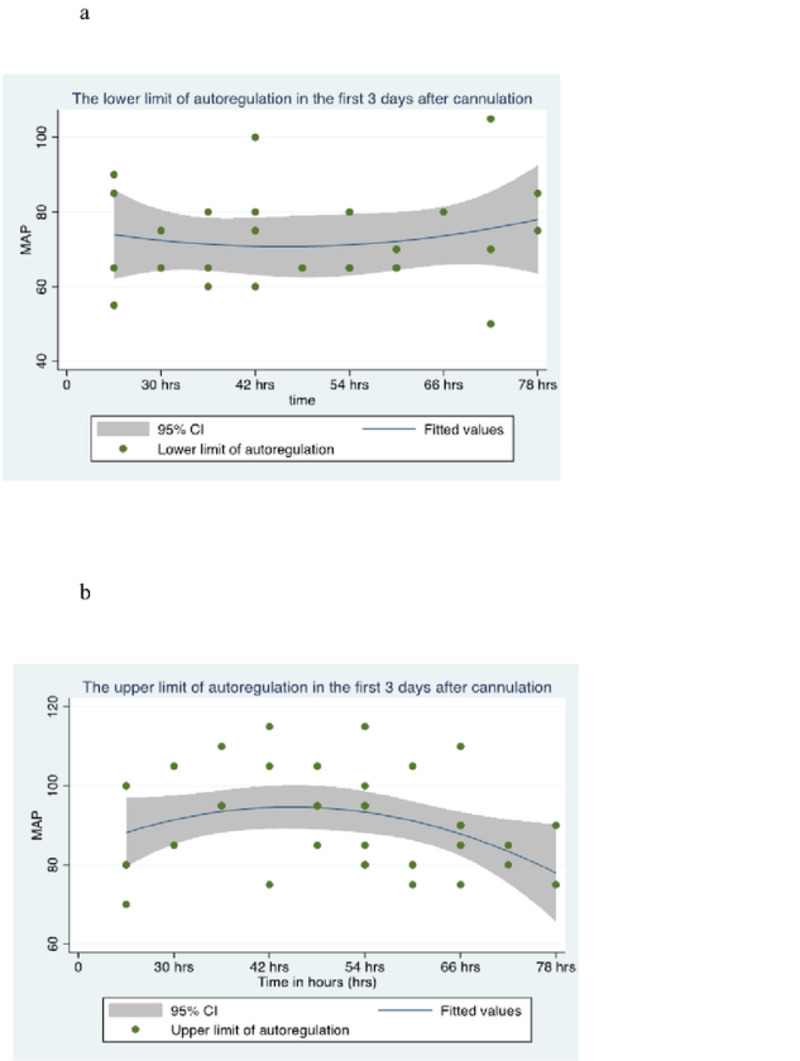
**a.** Scatter plot showing the lower limit of cerebral autoregulation with its 95% confidence intervals. **b.** Scatter plot showing the upper limit of cerebral autoregulation with its 95% confidence intervals.

**Figure 5.**
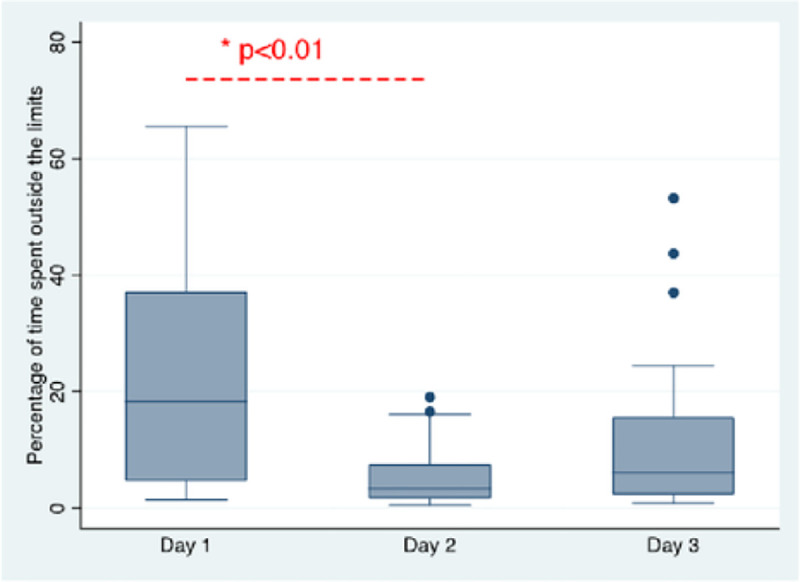
Boxplot showing the percentage of time the observed MAP was below LLA and above ULA over the first 3 days post-cannulation.

MAP_OPT_ was highly variable over the 72 hours of monitoring, ranging from 55 mmHg to 110 mmHg (Figure 3). The median MAP_OPT_ for the cohort was 75 mmHg (70–87.5 mmHg). The clinically observed median MAP was 77.8 mmHg (70.4–83.5 mmHg). LLA and ULA were identified in all but two patients over the duration of monitoring (Figures 4a and 4b). The LLA ranged from 50 to 105 mmHg, with a median LLA of 70 mmHg (65–80 mmHg). The ULA ranged from 70 to 115 mmHg, with a median ULA of 90 mmHg (80–100 mmHg). The percentage of time during which MAP_OBS_ was below the LLA and above the ULA was significantly higher on post-cannulation day 1 than on day 2 and 3 (18.2% vs 3.3%, p<0.01) (Figure 5). There was no difference in MAP_OPT_ between VA- and VV-ECMO patients.

### Neurological outcomes

**Figure 6.**
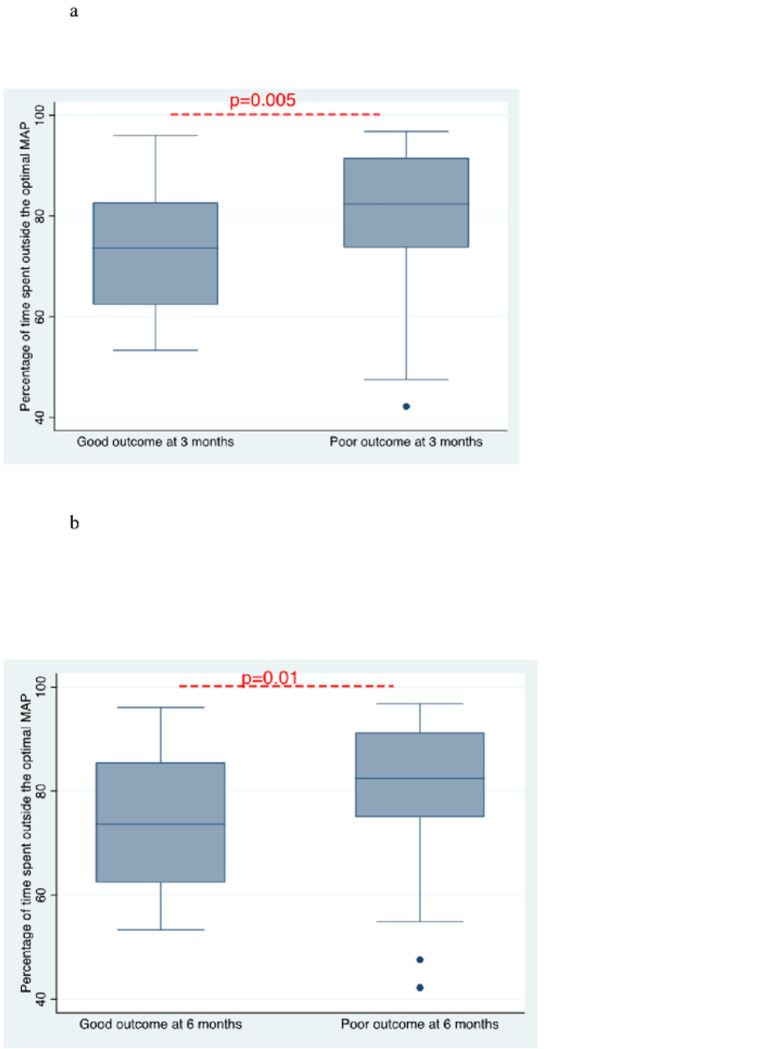
**a.** Boxplot comparing percentage of time the observed MAP was outside of the optimal MAP between patients who achieved a good functional neurologic outcome (mRS 0–3) and patients with a poor functional neurologic outcome (mRS 4–6) at 3 months. **b.** Boxplot comparing percentage of time the observed MAP was outside of the optimal MAP between patients who achieved a good functional neurologic outcome (mRS 0–3) and patients with a poor functional neurologic outcome (mRS 4–6) at 6 months.

Overall, 5 patients (33.3%) had at least one neurologic complication (Table 2). We found ischemic infarction in two patients, intracranial hemorrhage in two patients, and diffuse hypoxic-ischemic brain injury in one patient. One patient was in status epilepticus. One-third (n=5, 33.3%) had a good neurological outcome 3 months after hospital discharge. At 6 months, 40% (n=6) achieved a good neurologic outcome. Patients with a good neurologic outcome at 3 and 6 months spent significantly less time outside of MAP_OPT_ compared to patients with a poor neurologic outcome (74% vs 82%, *p*=0.01) (Figures 6a and 6b). AUC of patients was smaller in patients with a good neurologic outcome at 6 months, meaning the duration of time beyond limits of autoregulation and the magnitude of deviation from LLA and ULA were lower for the good neurologic outcome group than patients with the poor neurologic outcome (0.70 [0.22, 1.53] vs 3.02 [0.56, 7.7], *p*=0.5) (Supplemental Figure 1). However, this difference was not statistically significant.

## Discussion

Our cohort study is one of the first prospective, observational studies of its kind in adults ECMO patients. First, it showed that continuous monitoring of CA in adults supported by ECMO is feasible. Using routine, minimally invasive devices, we were able to start CA monitoring within a median 22 hours of ECMO initiation without causing any interference with the ECMO circuit or patient care. No adverse events associated with CA monitoring were reported. Second, data from this pilot study provide novel insights into the status of CA in patients on ECMO. We observed that mean COx significantly decreased between post-cannulation days 1 and 2, signaling improved CA after ECMO support. Similarly, the percentage of time during which clinically observed MAP values were outside of the CA limits decreased on day 2 compared to day 1. These findings are consistent with trends observed by Joram et al. in their pediatric ECMO population.^23^ Our pilot data also suggests CA may be more robust in patients supported by VV-ECMO than VA-ECMO. It is possible that CA is impaired in VA-ECMO patients due to their lack of blood flow pulsatility, which is believed to play a key role in the regulation of vascular tone by cerebrovascular endothelium.^24,25^ Another consideration is in our small cohort, cardiac arrest was only observed in VA-ECMO patients, and CA impairment following cardiac arrest has been reported in prior studies.^26,27^

Although CA was <0.3 for much of the monitoring time, the frequency of neurological complications remained high in our cohort, at 33%. Neurologic complications were identified after bedside CA monitoring had already ended in all except one patient. Given our sample size and limited duration of monitoring, we cannot draw any conclusions about whether impaired CA is associated with neurological complications in ECMO patients. This question warrants further investigation.

In this study, we explored the association between short and long-term neurological functional outcomes and time spent outside of the optimal MAP. We observed that more time spent in this “ideal” blood pressure was associated with having a good neurological outcome at 3 and 6 months. While severe hypo- and hyper-perfusion are known to cause acute brain injury, our data suggest that less dramatic deviations from an optimal perfusion target may have implications for functional neurologic outcomes months after decannulation. Another notable finding is we identified individualized optimal perfusion pressures for all patients and found the median optimal MAP to be 75 mmHg, which is 10 mmHg higher than perfusion targets recommended by current guidelines.^32,33^ Existing studies on CA after cardiac arrest have published similar findings, and the utility of adopting personalized perfusion goals on reducing acute brain injury or improving neurologic outcomes should be examined in future research.^26,34^

### Limitations

Our pilot study is exploratory in nature, limited by a small sample size and cohort heterogeneity. Patients had different modes and configurations of ECMO, as well as other mechanical circulatory support devices, which were not accounted for. Results were limited by the duration of CA monitoring, which was only a portion of the total time patients required ECMO support. There are also confounders that may have impacted our data, including body temperature, PCO_2_, PO_2_, depth of sedation, and unmeasured intracranial pressure. Finally, using COx as the basis for assessing CA in ECMO patients may draw some criticism, as the accuracy of this index has only been validated by a transcranial doppler-based method of assessing CA in adult patients undergoing cardiopulmonary bypass.^14^ Nevertheless, this pilot study serves as the foundation for our ongoing prospective study to investigate CA monitoring and physiology in ECMO patients.

## Conclusion

In adults supported by ECMO, it is feasible to monitor CA continuously at the bedside. CA improved over time, most significantly between post-cannulation days 1 and 2. CA was more impaired in patients supported by VA-than VV-ECMO. Spending less time outside of MAP_OPT_ was associated with achieving a good neurologic outcome at 3 and 6 months.

## Figures and Tables

**Table 1.** Characteristics of patients supported by veno-arterial and veno-venous extracorporeal membrane oxygenation.

	Whole cohort n=15	VA ECMO n=11	VV ECMO n=4

**Demographics**			
Age in Years, median [IQR]	57 [47–69]	57 [54–70]	47 [36–57]
Female, n (%)	5 (33.3%)	4 (36.4%)	1 (25%)
Race, n (%)			
White	11 (73.3%)	7 (63.6%)	4 (100%)
African American	3 (20%)	3 (27.3%)	0
Other	1 (6.7%)	1 (9.1%)	0
**Medical Comorbidities**			
Hypertension, n (%)	10 (66.7%)	9 (81.8%)	1 (25%)
Diabetes Mellitus	6 (40%)	6 (54.5%)	0
Atrial Fibrillation	4 (26.7%)	4 (36.4%)	0
Hyperlipidemia	9 (60%)	7 (63.6%)	2 (50%)
Coronary artery disease	7 (46.7%)	7 (63.6%)	0
Heart failure	7 (46.7%)	6 (54.5%)	1 (25%)
Cardiac arrest^1^	5 (33.3%)	5 (45.5%)	0
Chronic kidney disease	6 (40%)	5 (45.5%)	1 (25%)
Continuous renal replacement therapy	10 (66.7%)	7 (63.6%)	3 (75%)
SARS-CoV-2 Infection	4 (26.7%)	1 (9.1%)	3 (75%)
Sepsis	7 (46.7%)	3 (27.3%)	4 (100%)
**ECMO characteristics**			
Indications for ECMO, n (%)			
Cardiogenic shock	7	7	0
Post-cardiotomy	3	3	0
Respiratory failure	4	0	4
More than 1 indication^2^	1	1	0
ECMO duration, in days, median [IQR]	7 [4–13]	5 [3–10.5]	11 [9.5–13]
Cannulation site, n (%)			
Central	--	5 (45.5%)	--
Peripheral	--	6 (54.5%)	--
Other support devices^3^, n (%)			
Intra-aortic balloon pump	--	9 (81.8%)	--
Impella	--	1 (9.1%)	--
**Lab values immediately before cannulation**			
pH, median [IQR]	7.29 [7.24–7.36]	7.32 [7.2–7.38]	7.28 [7.26–7.3]
pCO_2_ (mmHg)	47 [36–60]	38 [35–48]	68 [58–76]
PaO_2_ (mmHg)	103 [71–278]	132 [95–299]	65 [54–72]
Lactate (mg/dL)	5.7 [3.3–10.4]	7.5 [4.8–11.8]	3.3 [2.4–6.9]
**Clinical outcomes**			
ICU length of stay, median [IQR]	13 [9.5–20]	14 [8–30]	11.5 [10–14]
Death at hospital discharge, n (%)	8 (53.3%)	6 (54.5%)	2 (50%)
Neurologic complications	5 (33.3%)	4 (36.4%)	1 (25%)
Good outcome (mRS 0–3) at 3 mo, n (%)	5 (33.3%)	4 (36.4%)	1 (25%)
Good outcome (mRS 0–3) at 6 mo, n (%)	6 (40%)	5 (45.5%)	1 (25%)

Abbreviations: ECMO: extracorporeal membrane oxygenation; IQR: interquartile range; mRS: Modified Rankin Score; n: number; VA: veno-arterial, VV: veno-venous

1 Cardiac arrest during index hospital admission.

2 One VA ECMO patient was cannulated for cardiogenic shock and respiratory failure.

3 One VA ECMO patient had both an intra-aortic balloon pump and Impella device.

**Table 2.** Patients supported by extracorporeal membrane oxygenation who developed neurologic complications and their outcome.

Case	Age years)	Sex	Primary diagnosis	Indication for ECMO	ECMO Mode	Cannulation site	Type of Neurologic Complication	Outcome at Hospital Discharge
1	58	F	Acute coronary syndrome	Cardiogenic shock	VA	Central	R posterior parietal lobe acute infarction, L tentorial subdural hematoma	Deceased
2	70	M	Acute respiratory distress syndrome	Respiratory failure	VV	-	Status epilepticus	Alive (mRS 5)^1^
3	37	F	Myocarditis	Cardiogenic shock	VA	Peripheral	L frontal lobe acute infarction	Alive (mRS 3)^2^
3	52	F	Non-ischemic cardiomyopathy	Cardiogenic shock	VA	Peripheral	L subdural hematoma L hemisphere, R frontal intraparenchymal and subarachnoid hemorrhage	Deceased
4	72	M	Acute coronary syndrome	Post-cardiotomy	VA	Central	Diffuse hypoxic-ischemic brain injury	Deceased

1 At 6 months after hospital discharge, patient 2’s mRS was 5.

2 At 6 months after hospital discharge, patient 3’s mRS was 2.

## Abbreviations

COx: cerebral oximetry index
ECMO: extracorporeal membrane oxygenation
LLA: lower limit of autoregulation
MAP: mean arterial pressure
ULA: upper limit of autoregulation
VA: veno-arterial
VV: veno-venous
